# The immune response to Covid-19 mRNA vaccination among Lymphoma patients receiving anti-CD20 treatment

**DOI:** 10.3389/fimmu.2024.1433442

**Published:** 2024-09-04

**Authors:** Edina Komlodi-Pasztor, Marta Escarra-Senmarti, Danielle A. Bazer, Aastha Bhatnagar, Carlos A. Perez Heydrich, Marcus Messmer, Richard F. Ambinder, Douglas E. Gladstone, Laura Clayton, Amy Goodrich, Laura Schoch, Nina Wagner-Johnston, Christopher J. VandenBussche, Peng Huang, Matthias Holdhoff, Maximillian Rosario

**Affiliations:** ^1^ Department of Oncology, Johns Hopkins University School of Medicine, Baltimore, MD, United States; ^2^ Department of Neurology, MedStar Georgetown University Hospital, Washington, DC, United States; ^3^ Department of Pathology, Division of Clinical Immunology, Johns Hopkins University School of Medicine, Baltimore, MD, United States; ^4^ Department of Hematology/Oncology, Fox Chase Cancer Center, Philadelphia, PA, United States; ^5^ Northwell Health Cancer Institute, New Hyde Park, NY, United States

**Keywords:** mRNA, COVID - 19, Tcell, vaccination, lymphoma, rituximab, CD20

## Abstract

The monoclonal antibody rituximab improves clinical outcome in the treatment of CD20-positive lymphomatous neoplasms, and it is an established drug for treatment of these cancers. Successful mRNA COVID-19 (SARS-CoV-2) vaccination is extremely important for lymphoma patients because they tend to be elderly with comorbidities which leaves them at increased risk of poor outcomes once infected by Coronavirus. Anti-CD20 therapies such as rituximab, deplete B-cell populations and can affect vaccine efficacy. Therefore, a knowledge of the effect of COVID-19 vaccination in this group is critical. We followed a cohort of 28 patients with CD20-positive lymphomatous malignancies treated with rituximab that started prior to their course of COVID-19 vaccination, including boosters. We assayed for vaccine “take” in the humoral (IgG and IgA) and cellular compartment. Here, we show that short-term and long-term development of IgG and IgA antibodies directed toward COVID-19 spike protein are reduced in these patients compared to healthy controls. Conversely, the robustness and breath of underlying T-cell response is equal to healthy controls. This response is not limited to specific parts of the spike protein but spans the spike region, including response to the conserved Receptor Binding Domain (RBD). Our data informs on rational vaccine design and bodes well for future vaccination strategies that require strong induction of T-cell responses in these patients.

## Highlights

- The B-cell response post Covid-19 mRNA vaccination is weaker among patients with CD-20 positive lymphomatous malignancies receiving or having received rituximab.
- Rituximab-treated lymphoma patients develop strong T-cell responses after Covid vaccination that is similar to healthy controls.

## Introduction

Viral infections can spread through populations with startling efficiency, necessitating public health measures to contain and control the spread. One of the most successful approaches involves vaccination which can generate protection from current and future pathogens of a similar nature thus reducing the likelihood of lethal outbreaks (1).

The respiratory virus SARS-CoV-2 first broke in 2019 and rapidly escalated to become a pandemic (2). In less than a year, both Pfizer-BioNTech (BNT162b2) and Moderna (mRNA-1273) developed mRNA vaccines that have proved protective against hospitalization and severe disease (3). To date, more than a billion doses of the mRNA vaccines have been administered. These and other vaccine makers targeted the original Wuhan strain SARS-CoV-2 spike protein (4).

The efficacy of vaccination is complicated by human diversity. Millions of patients are immunocompromised due to the presence of underlying conditions and/or specific medical intervention. For example, rituximab, a commonly used anti-CD20 therapy for B-cell associated auto-immune diseases and CD20-positive lymphomatous malignancies (5), enacts antitumor effects by direct signaling, cell-mediated cytotoxicity, and antibody-dependent cellular cytotoxicity (6). The depleted peripheral B-cell populations, crucial in humoral immunity for viral infections, might not recover for six to twelve months after treatment. Rituximab doses and schedules vary and depend on diagnosis and stage of the disease. Rituximab is used as monotherapy or in combination with chemotherapeutic regimens (e.g., Rituximab-CHOP). Upon infusion, rituximab-induced B-cell depletion occurs within days and lasts for several months after the last dose of treatment. (7–9). Also, side effects vary depending on the specific disease being treated; for example, an increase in regulatory T-cells has been reported post rituximab treatment in patients with systemic lupus erythematosus (10) but not in patients with rheumatoid arthritis (11).

The kinetics of CD20 depletion therapy in relation to vaccination is important because an intact and properly functioning B-cell population is critical for antibody formation. Further, the effect of rituximab is rapid, and it can take up to one year for this population to recover (5). Fortunately, with regard to SARS-CoV-2, both CD4+ and CD8+ T-cell responses have been correlated with better outcomes for both mildly infected and convalescent COVID-19 patients (12) including reduced disease severity (13, 14). A retrospective analysis comparing COVID-19 patients receiving rituximab for any indication demonstrated that only half developed neutralizing antibodies. Interestingly, there was no significant temporal difference between the final therapeutic infusion of the drug and COVID-19 diagnosis, suggesting that developing antibodies to the infection might not be paramount for recovery in these patients (15). Due to rituximab’s mechanism of action, it is abundantly clear that a substantial percentage of these patients are not developing a neutralizing antibody response to COVID-19 vaccination (16–21).

These discoveries prompt a larger clinical question about whether patients on rituximab therapy should be given vaccinations of any kind. If so, is there an ideal period after the final dose of anti-CD20 therapy that vaccinations should be administered or would it be more efficacious for the immunocompromised patient to pause B-cell depleting therapies for vaccinations, especially during a pandemic?

Given the gap in literature examining this question, we undertook a pooled peptide approach to better understand and evaluate the T-cell response to SARS-CoV-2 vaccination in a cohort of 28 lymphoma patients treated with rituximab during and after a COVID-19 vaccine regimen. The pooled peptide method delineates responses to vaccination missed by whole peptide or mass peptide assay techniques (22); it also reveals subdominant responses and can provide information about the breadth of coverage (23). Also, a time-course of response allows for comparison with antibody responses.

## Methods

### Trial design and patient selection

This observational clinical study investigated the immunological response of the SARS-CoV-2 vaccine in patients treated with anti-CD20 therapy. The study was approved by the Institutional Review Board (IRB-01-2020202013) and informed consent was obtained from all study participants.

Inclusion criteria were: (1) patients greater than 18 years of age, (2) a history of hematologic cancer, (3) received rituximab as part of cancer therapy prior to receiving SARS-CoV-2 vaccine, and (4) willingness to receive the SARS-CoV-2 vaccine. Exclusion criteria precluded patients who: (1) were not willing to receive the SARS-CoV-2 vaccine.

### Participants

Patients that met the eligibility criteria for this study and were interested in participating were referred to our study team by their providers.

### Study duration and interventions

The study duration was 18 months with a median and mean number of two blood draws ranging from 1-3. The median time from the initial blood draw to the final blood draw was 89.5 days and the mean time from the initial blood draw to the final blood draw was 83 days ranging from 1-184 days. Subjects underwent a series of standard blood tests, planned for: (1) between two SARS-CoV-2 vaccine doses - 2-3 weeks after the first dose and before the second dose, (2) post-vaccination dose - 2-3 weeks after the second dose, (3) follow-up #1 - 3 months after the second dose, (4) follow-up #2 - 6 months after the second dose, (5) follow-up #3 - 12 months after the second dose. If a patient chose to receive an additional dose of the COVID-19 vaccine (third dose), they underwent another blood test (6) after the additional vaccination - preferably 2-3 weeks after the third dose. Within a week of blood tests, the patients were screened for COVID-19 symptoms in person, by phone, or by video visit. Healthy control participants only provided a single blood draw after receiving the COVID-19 vaccine - 3 months after vaccination.

### Study measures

Both serum and PBMC were collected for anti-SARS-CoV-2 IgA and IgG antibody testing and T-cell responses. Blood tests were sent to the Clinical Immunology Department at Johns Hopkins Hospital for Antibody testing (Euroimmun).

#### Serum antibody testing

Lymphoma patients getting rituximab submitted serum samples longitudinally over the course of the study. Each sample was collected approximately 3-6 months post completion of the vaccination regimen. Participants submitted between 1-4 blood donations. For testing, some samples were available before vaccination as illustrated in **Table 1**. IgG and IgA antibody measurements were obtained using enzyme-linked immunosorbent assay (Euroimmun) with threshold values 1.23 and 11.0 (at saturation) for IgG and IgA, respectively. The units are arbitrary and are calculated in relation (optical density) to a humanized monoclonal antibody to SARS-CoV-2 spike protein.

**Table 1:** Study participant peak T cell response and spike specific humoral response to mRNA Covid vaccination given vaccine and booster timing in relation to rituximab treatment and disease recurrence.

Participant	Diagnosis	Days between last Rituximab treatmentand first vaccine	Sex	Age	Vaccine	Time between vaccine and blood draw/ Booster* if given	Peak T cell response	IgG/IgA^#^	Recurrence on Study
1	**CLL**	-307	F	65	Pfizer	204/183	141	Y	No
2	**MCL**	-277	M	64	Moderna	221/186	4092	nd	No
3	**BCL**	-93	M	71	Moderna	192	200	nd	No
4	**MZL**	-68	F	92	Moderna	211/209	112	nd	No
5	**MZL**	-67	F	68	Pfizer	120	1308	Y/Y	No
6	**DLBCL/PCNSL**	-42	M	60	Moderna	244	183	nd	No
7	**MCL**	-8	F	63	Moderna	210/176	1983	nd	No
8	**DLBCL/PCNSL**	-5	F	69	Pfizer	18	490	nd	No
9	**DLBCL/PCNSL**	13	M	62	Pfizer	50	4317	nd	No
10	**DLBCL**	15	F	75	Pfizer	53	110	nd	No
11	**DLBCL/PCNSL**	45	F	76	Pfizer	205/186	888	nd	No
12	BCL*	56	F	73	Moderna	158	353	nd	No
13	**CLL**	69	M	66	Pfizer	205	102	nd	No
14	**FL**	97	M	62	Moderna	46	3325	nd	No
15	**PT-LPD**	99	M	68	Moderna	48	435	Y/Y	Yes, Day 12
16	**HCL**	130	M	69	Pfizer	130	1131	Y	Yes, Day 270
17	**HCL**	137	M	58	Pfizer	269/182	754	Y/Y	No
18	**FL**	141	M	55	Pfizer	244	183	Y	Yes, Day 120
19	**CLL**	156	F	45	Moderna	229/199	138	Y/Y	No
20	**CLL**	156	M	66	Moderna	237	560	nd	No
21	**CLL**	166	F	55	Pfizer	134/119	443	nd	No
22	**HCL**	179	M	51	Moderna	213	1717	Y	No
23	**DLBCL**	202	M	53	Pfizer	64	2247	Y/Y	No
24	**BCL**	218	M	70	Pfizer	306	1000	Y/Y	No
25	**CLL**	266	M	68	Pfizer	18	490	nd	No
26	**MZL**	354	F	69	Pfizer	192	358	nd	No
27	**FL**	358	F	66	Pfizer	220/173	408	Y/Y	No
28	**MCL**	413	M	63	Pfizer	203/163	590	Y/Y	No
29	**Control**		M	74	Moderna	321	308	Y/Y	
30	**Control**		M	76	Moderna	287/75	1130	Y/Y	
31	**Control**		F	53	Pfizer	283/255	2143	Y/Y	
32	**Control**		F	65	Moderna	225	1070	Y/Y	
33	**Control**		M	60	Pfizer	216	178	Y/Y	

* Booster time (days) post the first vaccine if given.

# nd, Spike specific antibody not detected; Y, Spike specific IgG detected; Y/Y, Spike specific IgG and IgA detected.CLL, Chronic Lymphocytic Leukemia; MCL, Mantle cell Lymphoma; BCL, B-cell Lymphoma; MZL, Marginal Zone Lymphoma; DLBCL/PCNSL, Diffuse Large B-cell Lymphoma/Primary Central Nervous System Lymphoma; FL, Follicular Lymphoma; HCL, Hairy Cell Lymphoma; PTLPD, post-transplant lymphoproliferative disease.

#### Peripheral blood mononuclear cells isolation and storage

10mL of blood were collected from each patient or healthy control at sampling. Within 6 hours, PBMC were isolated by Ficol gradient centrifugation, washed twice and frozen in RPMI containing 10% DMSO. Samples were cooled to -80°C and then transferred to liquid nitrogen for storage.

#### CD4+ T cell isolation

Isolated PBMC were mixed with PBS washed CD8 Dynabeads (11147D) at 10^7^ cells/25ul beads at 4°C under continuous rotation for 20min. The remaining PBMC were isolated by magnet separation. The cells were washed and applied to an ELISPOT assay as described below.

#### Peptides

The following reagents were obtained through BEI Resources, NIAID, NIH: 1. Spike Glycoprotein (Stabilized) from SARS-Related Coronavirus 2, Wuhan-Hu-1 with C-Terminal Histidine and Twin- Strep® Tags, Recombinant from CHO Cells, NR-53937. 2. Receptor Binding Domain was obtained through BEI Resources, NIAID, NIH: Vector pCAGGS Containing the SARS Related Coronavirus 2, Beta Variant Spike Glycoprotein Receptor Binding Domain (RBD) Gene, NR 54007. 3. Peptide Array, SARS-Related Coronavirus 2 Spike (S) Glycoprotein, NR-52402. 13-17mer peptides (10aa overlap) spanning the length of the SARS-CoV-2 (USA-WA/2020) spike protein were diluted in high grade DMSO (Sigma) to yield a stock 50ug/ul. Peptides were combined into 9 pools and used in ELISPOT assay at 0.2ug/peptide/well.

#### ELISPOT

Mabtech ELISPOT kits were used with Millipore (Millipore-Sigma) MSAIP plates. Cells were plated at 200,000 cells/well in triplicate where possible and stimulated with 9 pools of peptides (BEI resources), whole spike protein (200ng/well) (BEI resources), and the RBD protein 200ng/well (Invivogen). Cells were incubated for 36 hours, and a well was considered positive if the average number of spots in the negative control was less than a test well. Phytohemagglutinin (Invitrogen) was used as the positive control. Spots were quantified in the Immune Monitoring Core at the Johns Hopkins Sidney Kimmel Comprehensive Cancer Center (NCI CCSG P30 CA006973) on a Autoimmun Diagnostika GmbH.

## Statistical analysis

In univariate comparisons between patients and controls, we used the two-sample Student t-test for continuous variables and Fisher’s exact test for categorical variables. Associations between two continuous variables were examined using Pearson correlation and visualized these relationships through scatter plots. Data were properly transformed to reduce skewness. To reduce data skewness, log-transformations were applied. In the multivariate analysis, mixed-effects model was used to compare patients and controls while accounting for correlations among repeated measures and adjusting for individual gender.

## Results

### Patient demographics

A total of 30 patients with CD20-positive lymphomatous neoplasms that met eligibility criteria were enrolled. Two participants later withdrew their consent. The median age was 66 (ranging from 45-92). 57.1% of the participants were male; 16 males and 12 females. Three were African American and 25 were Caucasian. Five patients had B-cell lymphoma (17.9%), 6 had chronic lymphocytic lymphoma (21.4%), 1 had diffuse large B-cell lymphoma (3.6%),3 had diffuse large B-cell lymphoma/primary central nervous system lymphoma (10.7%), 4 had follicular lymphoma (14.3%), 3 had hairy cell leukemia (10.7%), 3 had mantle cell lymphoma (10.7%), 2 had marginal zone lymphoma (7.1%), and 1 patient had post-transplant lymphoproliferative disease (3.6%). Seventeen patients received the Pfizer-BioNTech vaccination schema and 11 received the Moderna mRNA-1273 vaccine regimen (**Table 2**). In addition, 5 participants without diagnosis of CD20-positive lymphomatous malignancy were enrolled and served as healthy controls.

**Table 2:** Demographics of patients with CD20-positive lymphomatous malignancies and controls enrolled in this study with corresponding timing of vaccination from last rituximab dose, and peak -IFNγ response.

Patient Demographics		
Median Age	66 (range 45-92)	
Gender	Male	16 (57.1%)
	Female	12 (42.9%)
Race	African American	3 (10.7%)
	Caucasian	25 (89.3%)
Diagnosis	BCL	5 (17.9%)
	CLL	6 (21.4%)
	DLBCL	1 (3.6%)
	DLBCL/PCNSL	3 (10.7%)
	FL	4 (14.3%)
	HCL	3 (10.7%)
	MCL	3 (10.7%)
	MZL	2 (7.1%)
	Post-Transplant LPD	1 (3.6%)
Vaccine Brand	Pfizer	17 (60.7%)
	Moderna	11 (39.3%)

CLL, Chronic Lymphocytic Leukemia; MCL, Mantle cell Lymphoma; BCL, B-cell Lymphoma; MZL, Marginal Zone Lymphoma; DLBCL/PCNSL, Diffuse Large B-cell Lymphoma/Primary Central Nervous System Lymphoma; FL, Follicular Lymphoma; HCL, Hairy Cell Leukemia; PTLPD, post-transplant lymphoproliferative disease.

Eight patients continued to receive rituximab treatment after the first dose of Covid vaccination. Time between the first vaccination and the last dose of rituximab raged from 5 to 307 days. Ten patients and two healthy controls received a booster vaccination during the study. Recurrence of disease occurred in three patients. Twenty patients completed rituximab treatment prior to vaccination and the time between the last treatment and the vaccination ranged from 13 to 413 days (**Table 1**). There was no apparent association between rituximab administration, vaccine timing and peak IFNγ T cell response to the vaccine. The patients age at entry into the study ranged from 45 to 92 and no correlation was seen between patient age and peak IFNγ T cell response to the Covid-19 vaccine.

A comparison of peak T cell response to spike protein peptides shows that the peak T cell response is not statistically significantly associated with the number of days between the last rituximab administration and vaccine administration (Pearson correlation -0.0636, p-value 0.6261) (**Supplementary Table S1**). Also, the peak T cell response was not statistically significant with respect to age (Pearson correlation -0.2642, p-value 0.9314). A T-test comparing patients with controls, males with females, and type of vaccine (Moderna vs. Pfizer) did not show a statistical difference (**Table 3**) nor did restimulation of PBMC with whole spike protein or RBD when comparing patients with controls (**Table 3**). A similar comparison testing of the CD4^+^ T cells yielded the same results (**Supplementary Tables S2**, **S3**). Detectable Spike specific IgG levels were observed in 12 patients (42.8%) and IgA responses were detected in 9 patients (32.1%) all within the IgG positive subgroup.

**Table 3:** A comparison of patient with control total T cell response to individual peptide pools that cover the vaccine immunogen spike protein.

Table 3	Log 2 (Peak T cell Response	P value
Controls	9.4016+/- 1.4808	0.9144
Patients	9.3185+/- 1.7813	
Female	8.8178+/- 1.4824	0.1314
Male	9.7094+/- 1.8180	
Moderna	9.0905+/- 1.9610	0.3773
Pfizer	9.6576+/- 1.5254	
Controls (Whole Spike)	2.0635+/- 3.0704	0.5898
Patients (Whole Spike)	2.9267+/- 3.3783	
Controls (RBD)	5.5129+/- 3.2246	0.6073
Patients (RBD)	4.6555+/-3.2571	

### Pfizer-BioNTech and Moderna (aka Spikevax) vaccines induced little antibody response among patients taking rituximab

**Figure 1:**
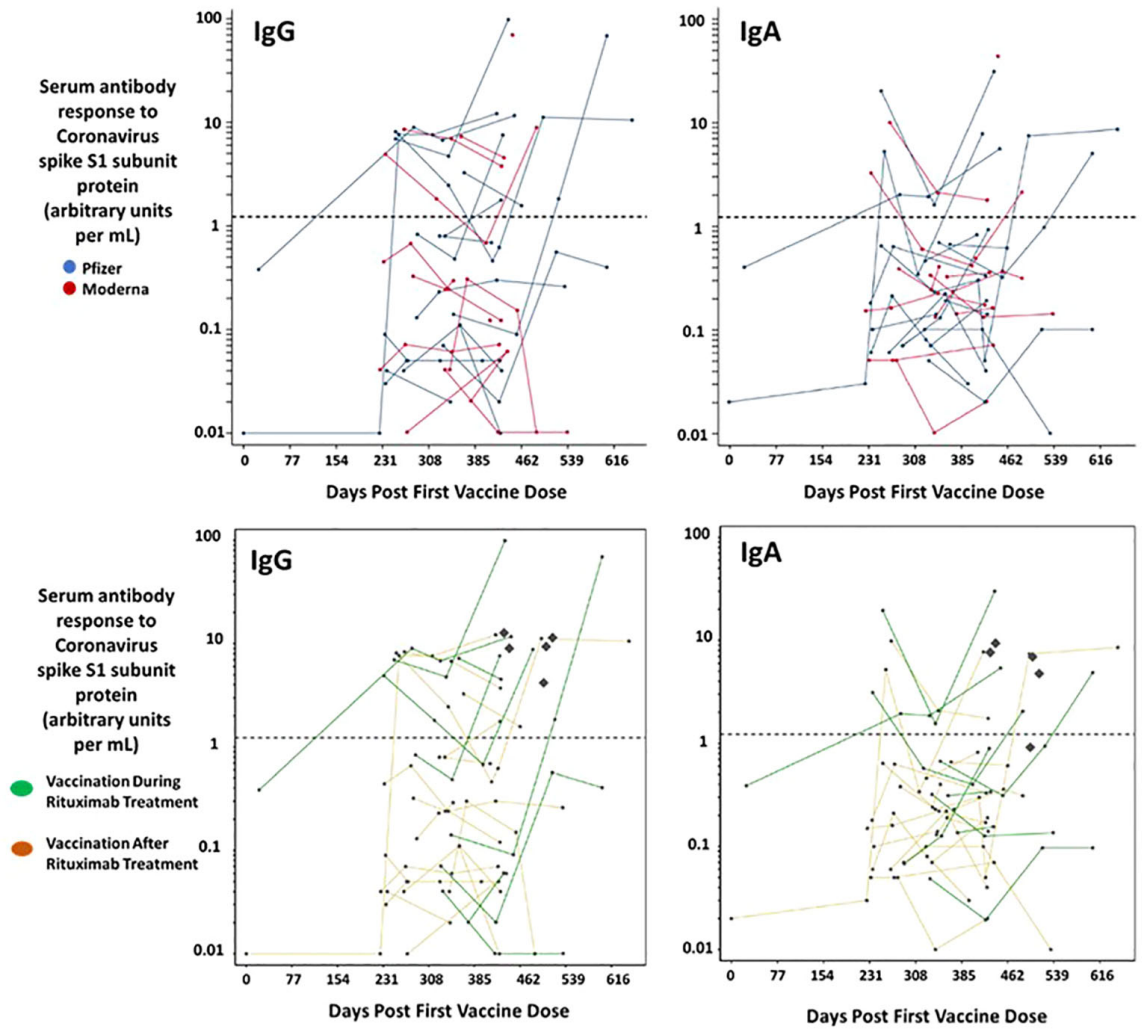
Serum immunoglobulin responses among patients with CD20-positive lymphoid malignancies receiving rituximab post mRNA vaccination for SARS-CoV-2. Serum samples were collected from 28 patients. Each dot represents a given patient sample and lines link patient samples over time. The horizontal dotted line represents the threshold at which an S1 spike specific antibody can be reported as positive by the Covid-19 immunoglobulin assay. The concentration of IgG and IgA S1 specific antibodies elicited post vaccination is similar both between males and females (not shown) and between Pfizer and Moderna vaccinees. The bottom panels delineate out the eight patients taking rituximab during (green) the study and those that stopped Rituximab before vaccination. No difference was observed. The Diamonds represent the Antibody response measured from the controls.

Serum was isolated and IgG responses to the S1 subunit of the SARS-CoV-2 spike protein were assayed. The limit of detection as determined by the assay was 1.14 (arbitrary units) with the majority of patients falling below 10 units. IgA responses to mRNA SARS-CoV-2 vaccination among our cohort were similarly low (**Figure 1**). In a previous study of healthy hospital workers, also carried out in the same laboratory with the same assay, most volunteer serum IgG units were 100 arbitrary units or greater; healthy volunteers both with and without prior SARS-CoV-2 infection maintained levels with a ratio averaging 4 and 7 times higher, respectively, at 200 days post vaccination (24). A comparison of IgG and IgA response to the Pfizer or Moderna vaccine showed no statistical difference nor did gender (**Supplementary Table S4**). It is important to note that during the study patients and controls may have been infected with Covid-19 and subsequently developed a subclinical response. We did not perform nucleocapsid antibody testing.

### Patient T cell response is similar to healthy controls post mRNA vaccination

**Figure 2:**
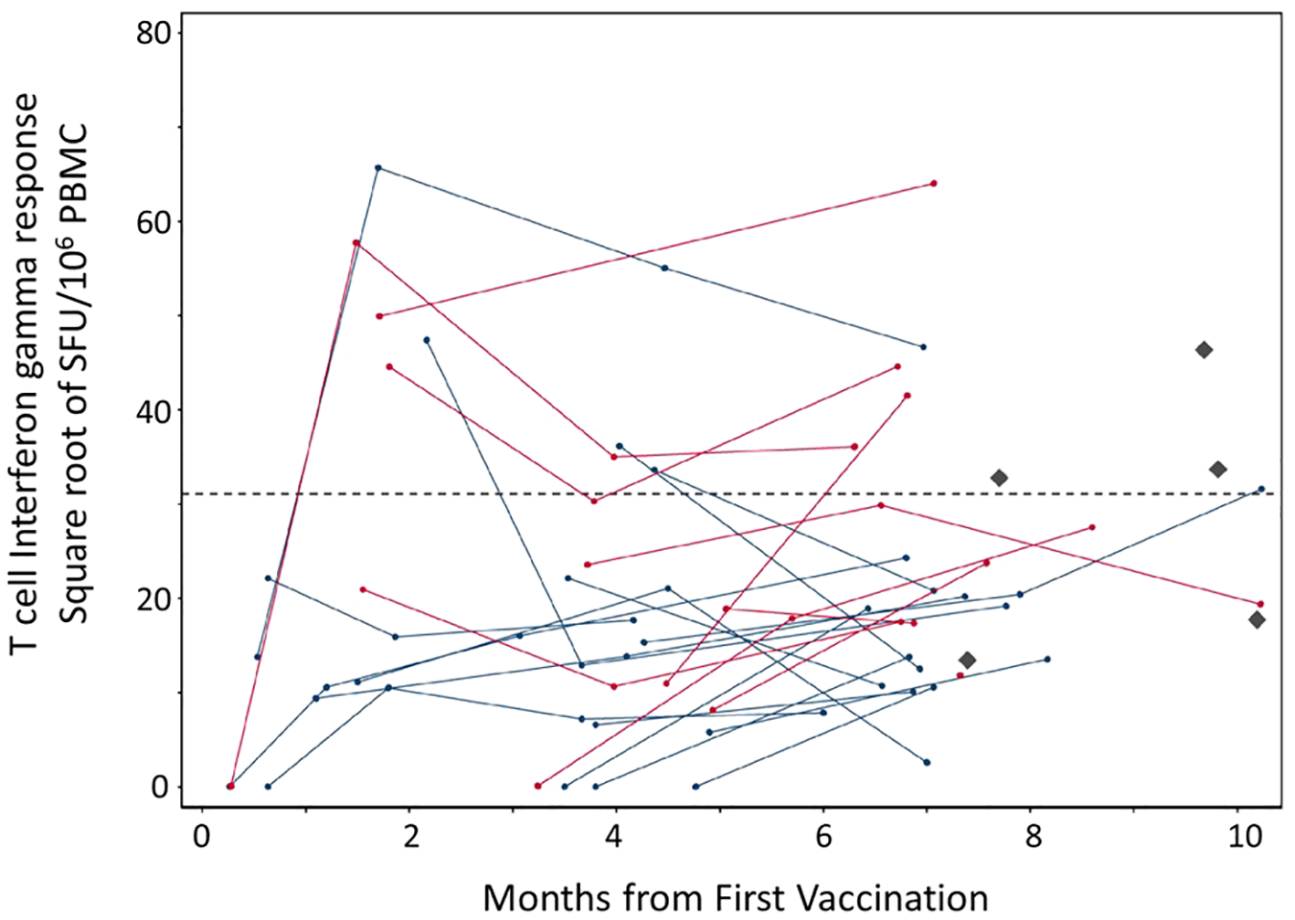
T cell responses to SARS-CoV-2 spike protein post mRNA vaccination among 28 patients with CD20-positive neoplasms receiving rituximab. PBMC were isolated from whole blood samples. Each dot represents a given patient sample and lines link patient samples over time. Total ELISPOT-Interferon gamma responses to the entire spike immunogen based on overlapping peptides that cover SARS-CoV-2 spike protein (Wuhan strain) in the Pfizer (blue) and Moderna (red) mRNA vaccines are illustrated. No difference between the two vaccines was observed. The Diamonds (black) represent healthy control responses and the dotted line represents that average of the healthy control T cell interferon gamma response.

**Figure 3:**
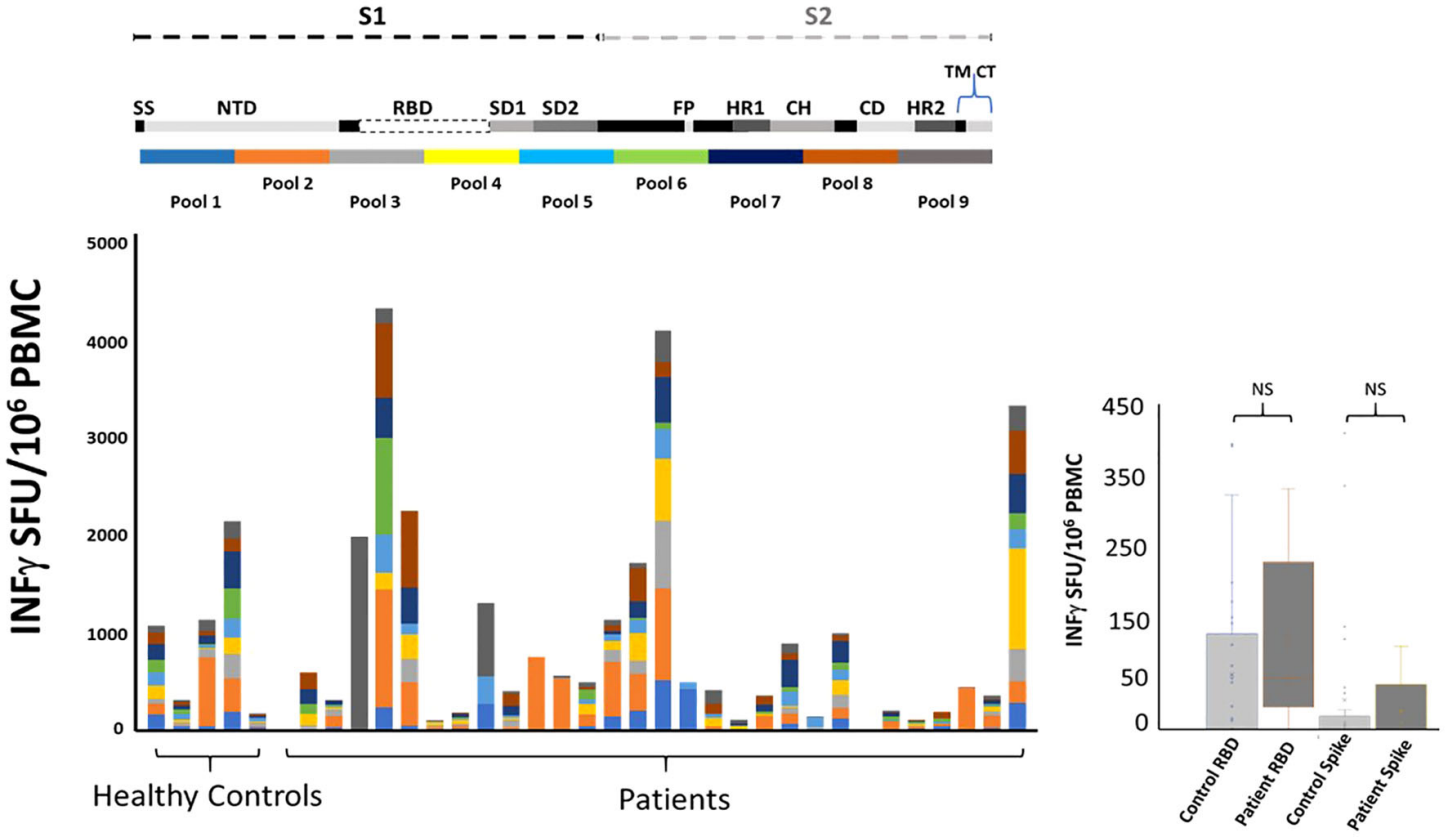
Immune responses to mRNA vaccination among patients with CD20-positive malignancy taking B cell depleting therapy (rituximab). Twenty-eight patients and five healthy controls underwent blood draws at approximately 3, 6- and 9-months post mRNA vaccination (standard dose and regimen Pfizer or Moderna; the healthy controls only had one blood draw). **Top:** Cartoon illustrating SARS-CoV-2 spike protein segments S1 and S2. The spike protein includes Signal sequence (SS), N-terminal Domain (NTD), Subdomain 1 and 2 (SD1, SD2), Fusion Peptide (FP), Heptad repeats (HR1 and HR2), Central Helix (CH), Connector Domain (CD), and Transmembrane Domain (TM) and cytoplasmic tail (CT). Ten pools of peptides (13-17mer 10aa overlap) were used to map T cell responses to the mRNA vaccines. **Bottom:** A comparison of total peak ELISPOT -IFNγ responses from all patients and healthy controls to the nine pools of peptide covering the vaccine immunogen. Note that all nine pools provide equal coverage. There is variation in the strength of the response but importantly there are individual preferences as to what regions of spike protein the T cells are targeting. **Right:** PBMC isolated from patients’ blood drawn at peak response post vaccination. ELISPOT responses to Receptor Binding Domain RBD and SARS-CoV-2 (Wuhan) spike protein (BEI resources and Invivogen) showed no significant difference with healthy control T cell responses. A log2(x+1) transformation was applied to the breadth (total of the 9 pools) to reduce skewness. We fitted data using a mixed effects model with group (patient or control) as a fixed effect and patient ID as random effect. The estimated fixed effect of the group is beta=-1.955 ± 1.488 (p=0.1930). Data are dichotomized by ≥40 or <40. We use two methods to compare each of these nine pools between cases (patients) and controls: (1) Fisher’s exact test and (2) logistic regression that adjusts for gender.

We measured total -IFNγ ELISPOT response to 9 pools of peptides overlapping the SARS-CoV2 spike protein. Patients fell into two broad categories (**Table 2**), those that were on rituximab at the time of vaccination and those that completed a final dose of the drug before being vaccinated. Blood draws for antibody testing approximated 3- and 6-months post initial vaccination. Kinetic T cell response remained stable for all the patients (**Figure 2**) during the course of the trial. 11 patients received one booster vaccine prior to blood draws (a third vaccination did not change T cell kinetics). There was a range in total T cell response, from no response to over 4000 SFU/10^6^ PBMC (**Figure 3**).

### The breadth of the cell mediated immune response is similar between patients and healthy controls post mRNA vaccination

It is well established that induction of broad robust T cell responses to viral infections leads to better outcomes (25–28). We compared T cell induction to mRNA SARS-CoV2 vaccination between healthy control and lymphoma patients with a history of rituximab administration. PBMC were tested for IFNγ response to nine pools of peptides covering the original Wuhan strain of spike protein. This approach uncovers T cell responses to epitopes not seen with whole protein stimulation. Our peptide pools consisted of 13-17mer amino acids with 10 aa. overlap to maximize coverage of all CD4^+^ and CD8^+^ T cell responses. The pools were equally divided across the spike protein and thus allowed us to match, by pool, the region of the spike protein each volunteer’ immune system was targeting post vaccination. The conserved region of spike protein “Receptor Binding Domain” RBD has been argued as a significant target for future SARS variants (29). We saw 3/5 volunteer control responses to the RBD region and 16/28 patient responses to the RBD region of the SARS genome. Similar to healthy controls there was variation in the strength and breath of response resulting in a range from over 200 to 2000 SFU/10^6^ PBMC among controls and from over zero to 4000 SFU/10^6^ PBMC among patients. There was no difference between patients and controls as cohorts when comparing whole spike protein or RBD protein as a stimulant (**Figure 3**). Individually comparing patients with controls for T cell response to the nine pools of peptides that represent the spike protein showed no difference (**Figure 3**; **Table 4**). Isolated responses from the CD4^+^ T cell subset showed comparable results (**Supplementary Table S5**).

**Table 4:** A comparison of patient with control total T cell response to individual peptide pools that cover the vaccine immunogen spike protein.

Table 4	Prop. Control	Prop. Patient	P value Fisher	beta regression	Std err regression	P value regression
Pool 1	0.8	0.4643	0.3353	-1.5437	1.1915	0.1951
Pool 2	0.6	0.7143	0.6269	0.5299	1.011	0.6002
Pool 3	0.6	0.4286	0.639	-0.7216	1.0369	0.4864
Pool 4	0.4	0.5	1	0.4526	1.0128	0.655
Pool 5	0.6	0.5714	1	-0.0939	1.0229	0.9269
Pool 6	0.6	0.3571	0.36	-1.0092	1.0153	0.3203
Pool 7	0.6	0.5357	1	-0.2682	0.9897	0.7864
Pool 8	0.6	0.4643	0.6562	-0.5424	0.9928	0.5848
Pool 9	0.6	0.4643	0.6562	-0.5424	0.9928	0.5848

Prop Control, proportion of controls with pool values 40 or above; Prop Patient, proportion of patients with pool values 40 or above. P value Fisher, p value from Fisher’s exact test. Beta regression, coefficient from logistic regression for cases (vs controls). stdev regression, standard error of Beta regression. P value regression, p value from the logistic regression.

## Discussion

In this study, we show that lymphoma patients on rituximab therapy generate T-cell responses to the spike protein of the Wuhan strain of SARS-CoV-2 similar to healthy controls. This T-cell response is induced despite a lack of antibody induction. Furthermore, we show that the response is as robust in healthy controls to both whole spike protein and the conserved RBD no matter the time post rituximab treatment or for that matter during treatment. The pooled peptide assay demonstrated that the breath of response is significant and targets similarly with healthy controls. We did not see significant differences between males and females nor between specific vaccines (Moderna vs Pfizer). Also, we did not see an age-related decline in T-cell activation post vaccination. Our data concurs with previous findings that total antibody induction of IgG and IgA to the spike protein of SARS-CoV-2 is greatly reduced in these patients.

B-cell depleting therapy has been shown to impact antibody responses to respiratory viruses, (30) but not cellular immune responses (31), which is consistent with their physiologic mode of action. Although the severity of Covid-19 infection in vaccinated patients receiving B-cell depleting therapy suggests worse outcomes (32), the data may be biased by co-morbidity. The situation is complicated by the diverse background of patients taking anti-CD20 treatments. Moreover, due to the changing nature of the pandemic the American College of Rheumatology is not making recommendations on Covid-19 vaccination (33). And, at the time of writing, the CDC recommends waiting six months post rituximab treatment before receiving a non-live vaccine due to suboptimal antibody response (2). In our study patients received multiple mRNA Covid-19 vaccinations in an attempt to generate protective antibodies. However, a time course analysis (**Figure 1**) demonstrated poor antibody responses in all but three patients that achieved specific S1 antibody levels comparable to healthy controls.

Vaccination in immunocompromised individuals represents a clinical dilemma, no matter which vaccine. For Covid-19, there is little data regarding vaccine timing and ‘take’ (34, 35). Our data sheds light on T-cell response regarding rituximab treatment timing. We show no correlation between T-cell -IFNγ response and rituximab administration.

Although emerging variants of Coronavirus have been effectively tackled by current vaccines, low frequencies of SARS-CoV-2 can evade neutralizing antibodies, whether elicited by prior infection or vaccination (36, 37). New variants alter transmissibility, reduce vaccine efficacy, reduce drug specific effectiveness, and reduce susceptibility to monoclonal antibody treatment (37). It is now becoming accepted that both cellular and humoral responses to Covid-19 contribute to viral control (38). Our findings together with others (20, 21, 39) argue for the development of T-cell dependent protective mechanisms against Coronavirus in these patients, as they are capable of inducing broad and robust T-cell responses. The effect of generating T-cell immunity should not be underestimated. Although antibodies can provide elevated levels of protection for six to seven months (40), combined with the emergence of infectious Omicron, it is the memory T-cell response that protects from severe disease (28). Our data demonstrates that patients can maintain T-cell responses over the course of the trial. Therefore, T-cell boosting therapeutics may bode well in this patient demographic.

Although cross-reactive T-cell responses have been found between upper and lower respiratory tract-infections, the effect of these responses remains unclear (41). Therefore, targeted vaccination remains a mainstay of protection against these highly pathogenic Coronaviruses.

Our study had limitations. These include a small sample size and heterogeneity of the patient population, with different diagnoses as well as different rituximab-containing regiments. In addition, Covid-19 infection could have occurred during the study period and although all patients received the full regiment of mRNA Covid-19 vaccines many received *ad-hoc* boosters. Nonetheless, the data provided are based on a prospective and carefully conducted sample collection, reflecting real-life clinical practice, and the observed signal of a robust T-cell response to Covid-19 vaccination, even in context of profound B-cell depletion in comparison with healthy controls.

These data contribute to a better understanding of vaccine responses in immunosuppressed patients, specifically those receiving CD20-targeted therapies. These therapies are not limited to cancer patients, as rituximab and similar CD20-targeted therapies are used for the treatment of autoimmune diseases, such as rheumatoid arthritis and multiple sclerosis. In addition, these findings may improve our understanding of the efficacy of other vaccines in this clinical setting and may guide further vaccine development.

## Data Availability

The original contributions presented in the study are included in graphs in the article/**Supplementary Material**. Further inquiries can be directed to the corresponding authors.
