# Supplementary material for: The immune response to Covid-19 mRNA vaccination among Lymphoma patients receiving anti-CD20 treatment
Source: Front Immunol. 2024 Sep 4;15:1433442. doi: 10.3389/fimmu.2024.1433442 (PMC11408186; doi:10.3389/fimmu.2024.1433442)
Supplement: Supplementary file 1 [file DataSheet1.pdf]

Supplementary Data

Table S1. Comparison of peak T cell response to spike protein peptides.

Variable	Pearson correlation with peak T cell	P value	95% confidence interval
Number of days between the last rituximab administration date and vaccine administration date.	-0.0636	0.6261	(-0.3737, 1.0000)
Age	-0.2642	0.9314	(-0.5161, 1.0000)

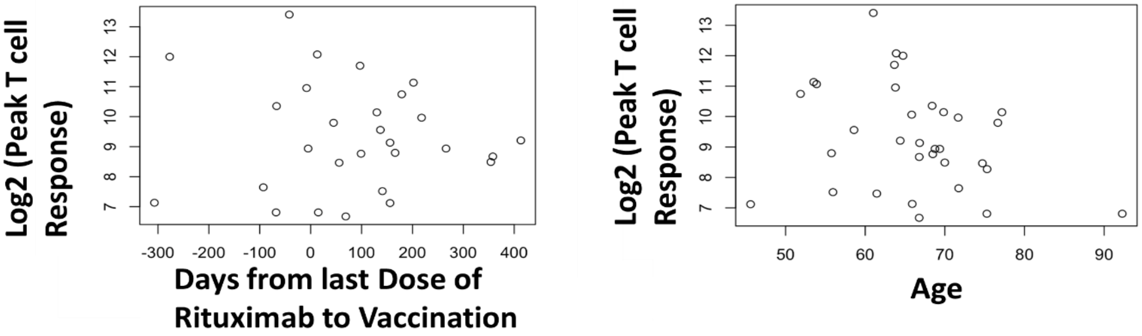

Left; Scatter plot comparing peak T cell INFg response to re-stimulation with Spike peptides and the number of days between the last Rituximab dose and first Covid-19 vaccination. **Right**; Scatter plot comparing peak T cell INFg response to re-stimulation with Spike peptides and patient age.

CD4 T cell Response

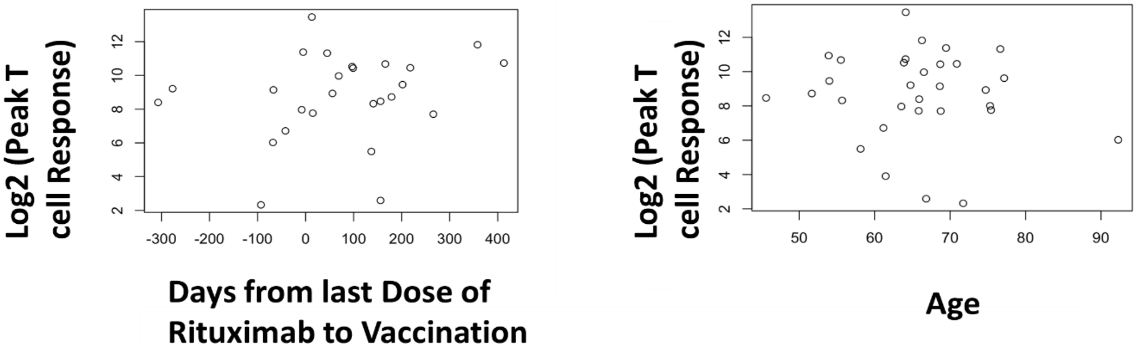

Left; Scatter plot comparing peak T cell INFg response to re-stimulation with Spike peptides and the number of days between the last Rituximab dose and first Covid-19 vaccination. **Right**; Scatter plot comparing peak T cell INFg response to re-stimulation with Spike peptides and patient age.

Table S2. Comparison of peak CD4 T cell response vs Timing of rituximab administration and Covid-19 vaccination.

Variable	Pearson correlation with peak T cell	P value	95% confidence interval
Number of days between the last rituximab administration date and vaccine administration date.	0.2170	0.1434	(-0.1218, 1.0000)
Age	-0.1019	0.7073	(-0.3911, 1.0000)

Table S3. Comparison of peak CD4 T cell response.

Table	Log 2 (Peak CD4 T cell Response)	P value
Controls	8.0358±2.6483	0.5921
Patients	8.7680±2.5970	
Female	9.2715±1.7886	0.2243
Male	8.2010±2.9890	
Moderna	9.4659±2.5349	0.0521
Pfizer	7.6591±2.3759	
Controls (Whole Spike)	4.0934±2.7596	0.9480
Patients (Whole Spike)	4.1885±3.4056	
Controls (RBD)	1.6951±2.8092	0.5301
Patients (RBD)	2.6274±3.1665	

Table S3. Comparison of Peak T cell response. Peak T cell responses are log2 transformed to reduce skewness. A two-sample t-test was used to compare peak T cell response between patients and controls, Females and Males, or Vaccine (Pfizer vs. Moderna) when PBMC are restimulated with overlapping peptides, and control vs. patient when PBMC are re-stimulated with whole spike protein, or with the Receptor Binding Domain protein.

Table S4. A comparison of antibody induction and analysis of correlation between IgA and IgG.

	Immunoglobulin	Variable	beta	Standard Error	P value
Vaccine	IgG	Pfizer	-0.5167	1.2857	0.6912
	IgA	Pfizer	0.0336	0.9416	0.9718
Gender	IgG	Male	0.965	1.2264	0.4389
	IgA	Male	0.5157	0.8991	0.5717

Table S4. Both IgG and IgA were log2 transformed to reduce skewness. Two mixed effects models were fitted to compare vaccine differences in IgG and IgA respectively, and two mixed effects models were fitted to compare gender differences in IgG and IgA respectively. In all 4 models, the random effect was the individual person. The results of the 4 models are summarized. No vaccine or gender difference comparing IgG or IgA induction was statistically significant. **Graph:** All IgA and IgG measures from blood samples drawn before the booster vaccination. For patients who received only one vaccine, all their blood samples were used. The Spearman correlation between IgA and IgG is R1=0.7457. These data are plotted in black "x" below.

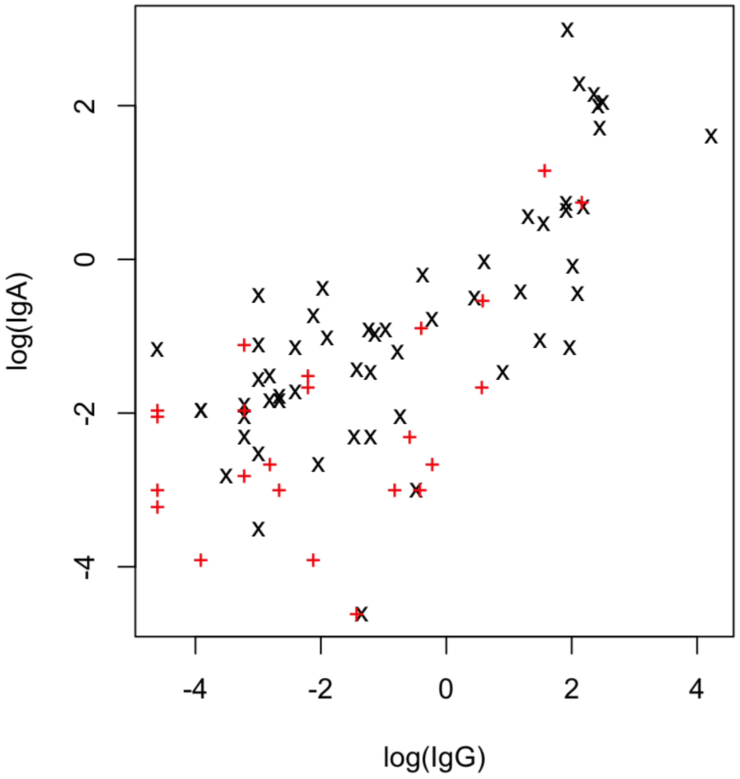

Individuals receiving sample: all IgA and IgG measures from blood samples drawn after the first vaccine. Only patients who received the second vaccine were included. The Spearman correlation between IgA and IgG is R=0.5043. These data are plotted in red "+" below.

The reason for using a Spearman correlation is because both IgA and IgG data are highly skewed.

Table S5. Comparison of patient with control response to individual peptide pools that cover the vaccine immunogen spike protein.

	Prop. Control	Prop. Patient	P value Fisher	beta regression	Std err regression	P value regression
Pool 1	0.2000	0.3462	1.0000	0.7475	1.1975	0.5325
Pool 2	0.4000	0.6538	0.3499	1.0414	1.0018	0.2986
Pool 3	0.2000	0.6154	0.1484	1.9628	1.2336	0.1116
Pool 4	0.2000	0.5385	0.3326	1.5438	1.1893	0.1943
Pool 5	0.2000	0.2692	1.0000	0.3808	1.2071	0.7524
Pool 6	0.4000	0.3077	1.0000	-0.4031	1.0074	0.6890
Pool 7	0.4000	0.6154	0.6254	0.8729	0.9996	0.3825
Pool 8	0.2000	0.3846	0.6310	0.9310	1.1925	0.4350
Pool 9	0.4000	0.2308	0.5831	-0.7953	1.0263	0.4384

Table S5. A comparison of patient with control response to individual peptide pools that cover the vaccine immunogen spike protein. A log2(x+1) transformation was applied to the breath (total of the 9 pools) to reduce skewness. We fitted data using a mixed effects model with group (patient or control) as a fixed effect and patient ID as random effect. The estimated fixed effect of the group is beta=-1.955±1.488 (p=0.1930). Data are dichotomized by ≥40 or <40. We use two methods to compare each of these nine pools between cases (patients) and controls: (1) Fisher's exact test; (2) logistic regression that adjusts for gender. Prop Control=proportion of controls with pool values 40 or above; Prop Patient=proportion of patients with pool values 40 or above. P value Fisher= p value from Fisher's exact test. Beta regression=coefficient from logistic regression for cases (vs controls). St.dev regression=standard error of Beta regression. P value regression = p value from the logistic regression.

Table S6. Description of treatment regimens for patients participating in the study.

ID#	Disease	Regimen Before Study	Regimen on Study	Covid Infection (day) by History	Death	Total Ritux cycles on study
1	**CLL**	ibrutinib+rituximab, R-Bendax2 cycles	Venetoclax	none	No	0
2	**MCL**	R-DHAP (substitute oxaliplatin for cisplatin), Autologous Hematopoietic Cell Transplantation, Maintenance Rituximab	Maintenance Rituximab every 8 weeks	none	No	6
3	**BCL**	Rituximab every week for 4 weeks, then rituximab every 3 months	Rituximab every 3 months	701	No	1
4	**MZL**	rituximab weekly x 8 cycles, then maintenance rituximab every 60days	Promacta	380	No	3
5	**MZL**	Rituximab induction, maintenance Rituximab every 90 days	Rituximab reinduction	none	No	4
6	**DLBCL/PCNSL**	none	Rituximab every 90 days	565	No	2
7	**MCL**	NCTNEA4181 - Arm B Bendamustine, Rituximab, High Dose Cytarabine and Acalabrutinib (BR/CR-A)	R-Benda+ Acalabrutinib, R-HiDAC + Acalabrutinib --> CR, Arm B-Rituximab only (wanted the drug locally)	none	No	5
8	**DLBCL/PCNSL**	HD MTX+ Rituximab + Temozolomide x 8 cycles, then HD MTX+Rituximab maintenance	HD MTX+Rituximab maintenance every month	none	No	7
9	**DLBCL/PCNSL**	None	R-CHOP and high-dose methotrexate, 6 cycles of J18168, Yescarta CAR T-cell infusion	239	537	4
10	**DLBCL**	HD MTX+Rituximab+Temozolomide, then maintenance Rituximab every 8 weeks	None	none	No	0
11	**DLBCL/PCNSL**	R-CHOP	None	none	No	0
12	**BCL***	Rituximab induction, Rituximab re-induction again, Rituximab re-induction again	None	none	No	0
13	**CLL**	Rituximab every 3 months	Rituximab every 3 months	620	No	4
14	**FL**	Rituxumab every 21 days	R-CHOP	none	No	4
15	**PT-LPD**	Pentostatin, rituximab induction, retuximab re-induction	None	61	No	0
16	**HCL**	Rituximab induction	None	none	No	0
17	**HCL**	HD MTX+ Rituximab every 3 weeks	HD MTX+ Rituximab every 3 weeks, TMZ. HD Ara-C, ASCT with BCNU/thiotepa	490	No	8
18	**FL**	rituximab induction, maintenance rituximab, re-induction and maintenance rituximab	Venetoclax	none	No	0
19	**CLL**	rituximab induction, rituximab maintenance every 90 days, re-induction and maintenance rituximab.	Venetoclax	636	No	0
20	**CLL**	Rituximab induction, single infusion of rituximab maintenance	Ibrutinib	546	No	0
21	**CLL**	2-CDA, pentostatin, rituximab induction, rituximab maintenance	None	504	No	0
22	**HCL**	R-CHOP, RICE, autologous peripheral blood stem cell transplant with BEAM conditioning	None	none	No	0
23	**DLBCL**	RCHOP x1 , DA-R-EPOCH x1 and IT Ara-C x2 and MR-CHOP x4	None	none	No	0
24	**BCL**	None	HD MTX+Rituximab every 3 weeks	Not known	No	9
25	**CLL**	Rituximab maintenance; Acalabrutinib 100 mg daily	Acalabrutinib 100 mg daily for 1 month on study, Venetoclax and IVIG	669	No	0
26	**MZL**	None	Rituximab induction x4 doses, IVIG	none	No	4
27	**FL**	R-Bendamustine 6 cycles	None	Before vaccine	No	0
28	**MCL**	R-Benda, R-DHAOx4 cycles, Ibrutinib/Veneteloclax, NMA Mismatch Unrelated Donor BMT, Promacta	Promacta	none	Mar-01	0
